# High risk clear cell renal cell carcinoma microenvironments contain protumour immunophenotypes lacking specific immune checkpoints

**DOI:** 10.1038/s41698-023-00441-5

**Published:** 2023-09-11

**Authors:** Arti M. Raghubar, Nicholas A. Matigian, Joanna Crawford, Leo Francis, Robert Ellis, Helen G. Healy, Andrew J. Kassianos, Monica S. Y. Ng, Matthew J. Roberts, Simon Wood, Andrew J. Mallett

**Affiliations:** 1 Kidney Health Service, Royal Brisbane and Women’s Hospital, Herston, QLD Australia; 2 Conjoint Internal Medicine Laboratory, Chemical Pathology, Pathology Queensland, Health Support Queensland, Herston, QLD Australia; 3 Faculty of Medicine, University of Queensland, Brisbane, QLD Australia; 4 Anatomical Pathology, Pathology Queensland, Health Support Queensland, Herston, QLD Australia; 5 Institute for Molecular Bioscience, University of Queensland, Brisbane, QLD Australia; 6 Faculty of Health, Charles Darwin University, Darwin, NT Australia; 7 QCIF Facility for Advanced Bioinformatics, Institute for Molecular Bioscience, The University of Queensland, Brisbane, QLD Australia; 8 Department of Urology, Princess Alexandra Hospital, Brisbane, QLD Australia; 9 Nephrology Department, Princess Alexandra Hospital, Woolloongabba, QLD Australia; 10 Department of Urology, Royal Brisbane and Women’s Hospital, Brisbane, QLD Australia; 11 Centre for Clinical Research, The University of Queensland, Brisbane, QLD Australia; 12 College of Medicine & Dentistry, James Cook University, Townsville, QLD Australia; 13 Department of Renal Medicine, Townsville University Hospital, Townsville, QLD Australia

**Keywords:** Renal cell carcinoma, Cancer genomics

## Abstract

Perioperative immune checkpoint inhibitor (ICI) trials for intermediate high-risk clear cell renal cell carcinoma (ccRCC) have failed to consistently demonstrate improved patient outcomes. These unsuccessful ICI trials suggest that the tumour infiltrating immunophenotypes, termed here as the immune cell types, states and their spatial location within the tumour microenvironment (TME), were unfavourable for ICI treatment. Defining the tumour infiltrating immune cells may assist with the identification of predictive immunophenotypes within the TME that are favourable for ICI treatment. To define the immunophenotypes within the ccRCC TME, fresh para-tumour (pTME, *n* = 2), low-grade (LG, *n* = 4, G1-G2) and high-grade (HG, *n* = 4, G3-G4) tissue samples from six patients with ccRCC presenting at a tertiary referral hospital underwent spatial transcriptomics sequencing (ST-seq). Within the generated ST-seq datasets, immune cell types and states, termed here as exhausted/pro-tumour state or non-exhausted/anti-tumour state, were identified using multiple publicly available single-cell RNA and T-cell receptor sequencing datasets as references. HG TMEs revealed abundant exhausted/pro-tumour immune cells with no consistent increase in expression of *PD-1, PD-L1* and *CTLA4* checkpoints and angiogenic genes. Additional HG TME immunophenotype characteristics included: pro-tumour tissue-resident monocytes with consistently increased expression of *HAVCR2* and *LAG3* checkpoints; an exhausted CD8^+^ T cells sub-population with stem-like progenitor gene expression; and pro-tumour tumour-associated macrophages and monocytes within the recurrent TME with the expression of *TREM2*. Whilst limited by a modest sample size, this study represents the largest ST-seq dataset on human ccRCC. Our study reveals that high-risk ccRCC TMEs are infiltrated by exhausted/pro-tumour immunophenotypes lacking specific checkpoint gene expression confirming that HG ccRCC TME are immunogenic but not ICI favourable.

## Introduction

Clear cell renal cell carcinoma (ccRCC) accounts for the majority of kidney cancer-related deaths due to the presence or development of metastatic disease^1,2^. Approved first-line systemic therapy for metastatic ccRCC includes immunotherapies such as immune checkpoint inhibitors (ICIs) that serve to reactivate anti-tumour immune responses^1,3–10^. However, the final results from adjuvant ICI trials for localised ccRCC have failed to demonstrate an improvement in survival outcomes^11–13^. The efficacy of perioperative ICIs within intermediate high-risk ccRCC patients has been assessed in randomised phase III clinical trials^4,5,14–17^. These perioperative (or neoadjuvant) ICIs are aimed at priming the anti-tumour immune response without delaying surgical intervention^15,17^. A recent neoadjuvant nivolumab trial for localised ccRCC reported no delays in surgery^16^. However, due to the short-term follow-up in this trial, it is uncertain if an anti-tumour immune response was initiated by earlier ICI treatment.

**Fig. 1:**
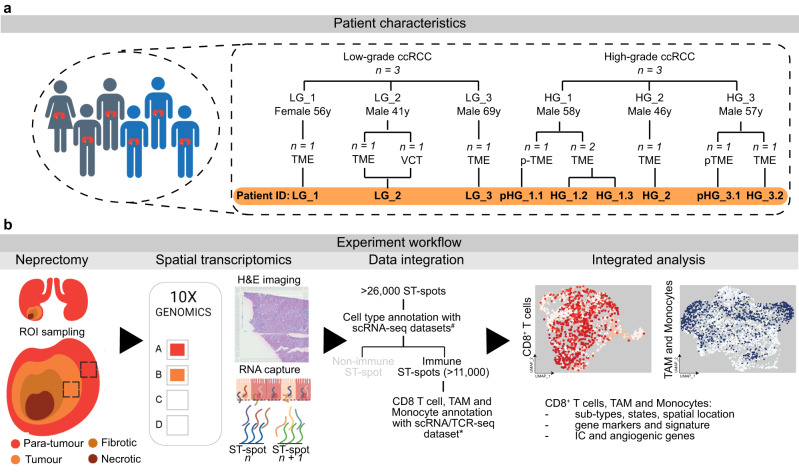
Schematic of patient characteristics and experiment workflow. The patient characteristics (**a**) of the six ccRCC patients (*n* = 3 LG and *n* = 3 HG) include: patient LG_2 with a vena cava thrombus (VCT) for which we collected primary tumour microenvironment (TME) and thrombi separately but processed in the one capture array for ST-seq; patient HG_1 that we collected and processed tissues from para-TME (pTME) and TME; and patient HG_3 that we collected tissues from pTME and TME. For this experimental workflow (**b**), ten tissue regions were sampled from pTME, TME and VCT that excluded fibrotic and necrotic regions. ST-seq was completed using 10x Genomics Visium Gene Expression microarrayed glass slides with unique spatially barcoded ST-spots that captured the mRNA released from the overlaying thin ccRCC tissue sections. Annotation of immune ST-spots was completed with data integration of six published single-cell RNA-sequencing (scRNA-seq) datasets. Further immune cell sub-typing was completed with a scRNA and T-cell receptor (TCR) sequencing dataset. Integrated analysis was completed on CD8^+^ T cells, TAM and monocytes.

The above-mentioned conflicting outcomes from ICI trials suggest that the identification of favourable ICI tumour microenvironments (TME) is an urgent unmet need for personalising oncology in high-risk ccRCC patients. Defining the TME-specific CD8^+^ T cells, tumour associate macrophages (TAM) and monocytes may clarify the precise immunophenotypes within the intermediate high-risk TME. The advancement within transcriptomics methodologies makes it feasible to profile the ccRCC tumour infiltrating immunophenotypes—defined here as the immune cell types, exhausted/pro-tumour or non-exhausted/anti-tumour states, and their spatial location within an individual patient’s TME (Table 1). This study profiled the immunophenotypes within the ccRCC TME from six consenting patients, using Visium ST-seq (10x Genomics) (Fig. 1 and Table 2). We hypothesise that the intermediate high risk (i.e., high-grade) ccRCC TME is composed of heterogenous exhausted/pro-tumour immunophenotypes and variable IC gene expression. Therefore, both the immunophenotype and IC gene expression for individual ccRCC patients must be profiled for the clinical targeting of ICI favourable TME.

**Table 1:** Delineation of CD8^+^ T cells, TAM and monocytes.

Abbreviation	Cell type	Cell sub-type	State
CD8^+^ tissue-resident	CD8^+^ T cells	Tissue-resident	Non-exhausted
CD8^+^ NK-like		NK-like	Non-exhausted
CD8^+^ exhausted		Exhausted	Exhausted
CD8^+^ exhausted IEG		Exhausted immediate-early genes	Exhausted
CD8^+^ proliferative		Proliferative	Exhausted
TAM ISG^int^	Tumour-associated macrophages (TAM)	Interferon signalling genes intermediate expression (ISG^int^)	Anti-tumour
TAM HLA^hi^		Human leucocyte antigen DR (HLA-DR) high expression (HLA^hi^)	Pro-tumour
TAM HLA^int^		HLA-DR intermediate expression (HLA^int^)	Pro-tumour
TAM ISG^hi^		ISG high expression (ISG^hi^)	Pro-tumour
Tissue-resident monocytes	Monocytes	Tissue-resident monocytes	Pro-tumour

**Table 2:** Clinical characteristics of ccRCC patients.

	Low-grade (LG) ccRCC			High-grade (HG) ccRCC				
Patient	LG_1	LG_2	LG_3	HG_1		HG_2	HC_3	
Sample region/s	TME (*n* = 1)	TME (*n* = 1) and VCT (*n* = 1)	TME (*n* = 1)	pTME (*n* = 1)	TME (*n* = 2)	TME (*n* = 1)	pTME (*n* = 1)	TME (*n* = 1)
Patient ID	LG_1	LG_2	LG_3	pHG_1.1	HG_1.2 HG_1.3	HG_2	pHG_3.1	HG_3.2
Sex, age (y)	Female, 55–60	Male, 40–45	Male, 65–70	Male, 55–60		Male, 45–50	Male, 55–60	
Tumour size, grade, stage	35 mm, G1, pT1a	140 mm, G1, pT3b	70 mm, G1, pT1b	125 mm, G4, pT3a		90 mm, G4, pT3a	18 mm, G3, pT1a	
HTN	No	No	Yes	Mild (nil treatment)		Yes	No	
Diabetes	No	No	No	No		No	No	
Smoker	No	No	No	Ex-smoker		Ex-smoker	Ex-smoker	
Obesity	No	No	Yes	No		No	Yes	
Other comments	No-coexisting pathology in normal kidney	Tumour thrombi extending into inferior vena cava	No lymph nodes in the sample	Metastases in pleura (diagnosed 2 weeks post nephrectomy)		No-coexisting pathology in normal kidney	2008 RCC right kidney 2017 RCC left kidney	
Treatment	Laparoscopic left radical nephrectomy	Open right nephrectomy	Laparoscopic right radical nephrectomy	Open right nephrectomy. Ipilimumab/ nivolumab C1 (ceased due to colitis + dermatitis) Nivolumab C5 (ceased due to colitis) SBRT to left pleural-based metastasis		Laparoscopic radical left nephrectomy	2008 Right nephrectomy 2017 Partial left nephrectomy 2021 Redo partial left nephrectomy	
Last follow-up (dd/mm/yyyy), outcome.	04/11/2021, No evidence of local recurrence (NELR), No new metastatic disease (NNMD)	20/08/2021, NELR, NNMD	15/12/2021, NELR, NNMD	28/01/2022, NELR, NNMD		18/06/2021, NELR, NNMD	08/11/2021, NELR, NNMD	
Whole exome sequencing: Gene (mutation, variant allele frequency)	No	No	No	Yes: VHL (Leu135Ter, 13.4% and Pro138Leu, 13.2%), BAP1 (Ala644CysfsTer4, 12.3%) TET2 (Leu179GLYfsTer5, 11.1%) and MTOR (Met304Leu, 4.8%)		No	No: genes tested (BAP1, FH, FLCN, MET, PTEN, SDHB, SDHC, SDHD, TSC1, TSC2, VHL) – no pathologic variants, likely pathogenic variants or variants of unknown significance detected	

## Results

### Pro-tumour immune cell signatures appear restricted to HG ccRCC TMEs

**Fig. 2:**
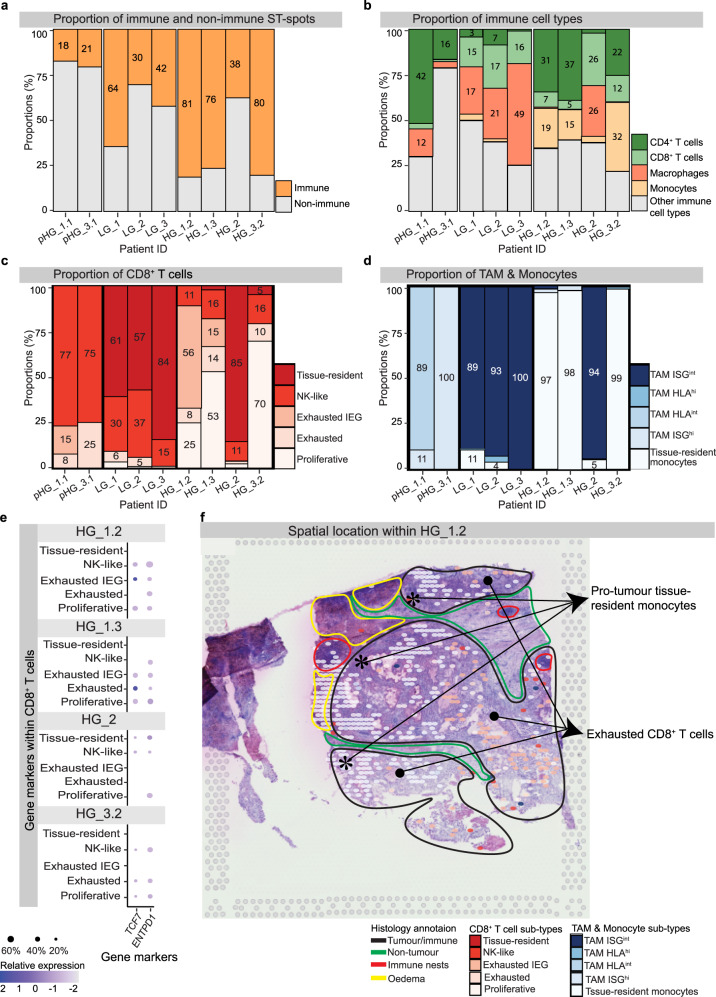
Proportions of CD8^+^ T cell, TAM and monocyte sub-types within the ccRCC TME. The proportion of immune ST-spots (**a**) increased with ccRCC grade. Further immune cell typing of the immune ST-spots revealed higher proportions of T cells (CD8^+^ and CD4^+^ T cells) in pTME and HG TME and macrophages in LG TME (**b**). Within the identified proportion of CD8^+^ T cellS, finer sub-typing (**c**) identified abundant non-exhausted CD8^+^ NK-like and tissue-resident cells in the pTME, LG TMEs and HG_2 TME. In contrast, exhausted CD8^+^ T cells were identified in HG_1 and HG_3 TMEs. TAM and monocyte sub-typing (**d**) identified non-exhausted TAM ISG^int^ cells within all LG and HG_2 TMEs. Abundant exhausted tissue-resident monocytes were identified in HG_1 and HG_3 TMEs. Similarly, exhausted TAM HLA^int^ and TAM ISG^hi^ were identified in pHG_1.1 and pHG_3.1. The proportion of CD8^+^ T cell (**c**), TAM and monocyte (**d**) sub-types are based on the immune cell types (**b**) and immune ST-spots (**a**) proportions. Within the HG TMEs, we further investigated heterogeneity within CD8^+^ T cells (**e**). For the HG TMEs, we found sub-populations within the exhausted CD8^+^ T cells expressing progenitor (*TCF7*) or immunomodulatory (*ENTPD1*) genes. Spatial mapping of the variable proportions of CD8^+^ T cells, TAM and monocytes (**f**) within HG ccRCC TMEs demonstrated abundant pro-tumour tissue-resident monocytes surrounding the exhausted CD8^+^ T-cell sub-types within defined tumour/immune admixed regions, as presented within the representative HG_1.2 TME.

Overall, the proportion of immune ST-spots significantly increased (*p*-value 2.9^e-10^) from para-tumour (pTME), low-grade (LG) and high-grade (HG) TMEs (Fig. 2a). The highest proportion of CD4^+^ T cells were found within pTME and HG TMEs (p-value 1.8^e-5^) and macrophages within LG TMEs (*p*-value 0.006) (Fig. 2B and Supplementary Table 1). Further CD8^+^ T-cell sub-typing (Fig. 2c) revealed highest CD8^+^ tissue-resident cells within LG TMEs (*p*-value 3.1^e-3^). Conversely, the highest exhausted CD8^+^ proliferative cells were found within HG TMEs (*p*-value 8.9^e-3^). TAM and monocyte sub-typing (Fig. 2d) revealed highest TAM with interferon signalling genes intermediate expression (TAM ISG^int^) within the LG TMEs (p-value 9.5^e-13^). However, the highest tissue-resident monocytes were found mainly within HG TMEs (p-value 7.3^e-11^). Interestingly, the CD8^+^ T cells, TAM and tissue-resident monocytes identified within the LG TMEs were mirrored within the HG_2 TME. The proportion of immune cell sub-types was based on the immune cell types (Fig. 2b) and immune ST-spots (Fig. 2a). Hence, the final proportions of individual immune cell sub-types are very low within the TME which is composed of non-immune and immune ST-spots.

Within the identified CD8^+^ T-cell sub-types, we found low expression of progenitor transcription factor 7 (*TCF7*) and immunomodulatory marker ectonucleoside triphosphate diphosphohydrolase 1 (*ENTPD1*). Nonetheless, the *TCF7* revealed the highest relative expression within sub-populations of CD8^+^ exhausted immediate-early genes (CD8^+^ exhausted IEG) and CD8^+^ exhausted T cells in HG_1.2 and HG_1.3 TMEs (Fig. 2e and Supplementary Table 2). The *ENTPD1* revealed the highest relative expression within sub-populations of CD8^+^ proliferative T cells in HG_1.3 and HG_3.2 TMEs, and CD8^+^ tissue-resident T cells within HG_2 TMEs (Fig. 2e and Supplementary Table 2). Within the identified myeloid sub-types a higher relative expression of triggering receptor expressed on myeloid cells 2 (*TREM2*), a known marker for recurrence, was identified within a larger proportion of pro-tumour TAM sub-types in HG_3 TME; whilst an absence of immunosuppression markers human leucocyte antigen G (*HLA-G)* and cysteine protease cathepsin S (*CTSS*)^18,19^ was observed within TAM ISG high expression (TAM ISG^hi^) in HG_3 and HG_2 TME, respectively (Supplementary Fig. 1).

### Tissue-resident monocytes surround the exhausted CD8^+^ T cells and express novel immune checkpoint (IC) genes *HAVCR2* and *LAG3*

To establish the spatial location of the immune cells within the TMEs, we mapped CD8^+^ T cells, TAM and monocytes back to their corresponding tissue sections. We found tissue-resident monocytes surrounded the exhausted CD8^+^ T cells within tumour/immune admixed regions of the HG_1.2 TME (Fig. 2f). Unfortunately, the other TMEs did not contain a histomorphologically distinct and large non-tumour region surrounding the tumour/immune admixed region. Nonetheless, HG_1.3 and HG_3.2 TMEs revealed exhausted CD8^+^ T-cell sub-types and pro-tumour tissue-resident monocytes within the tumour/immune admixed regions (Supplementary Fig. 2).

**Fig. 3:**
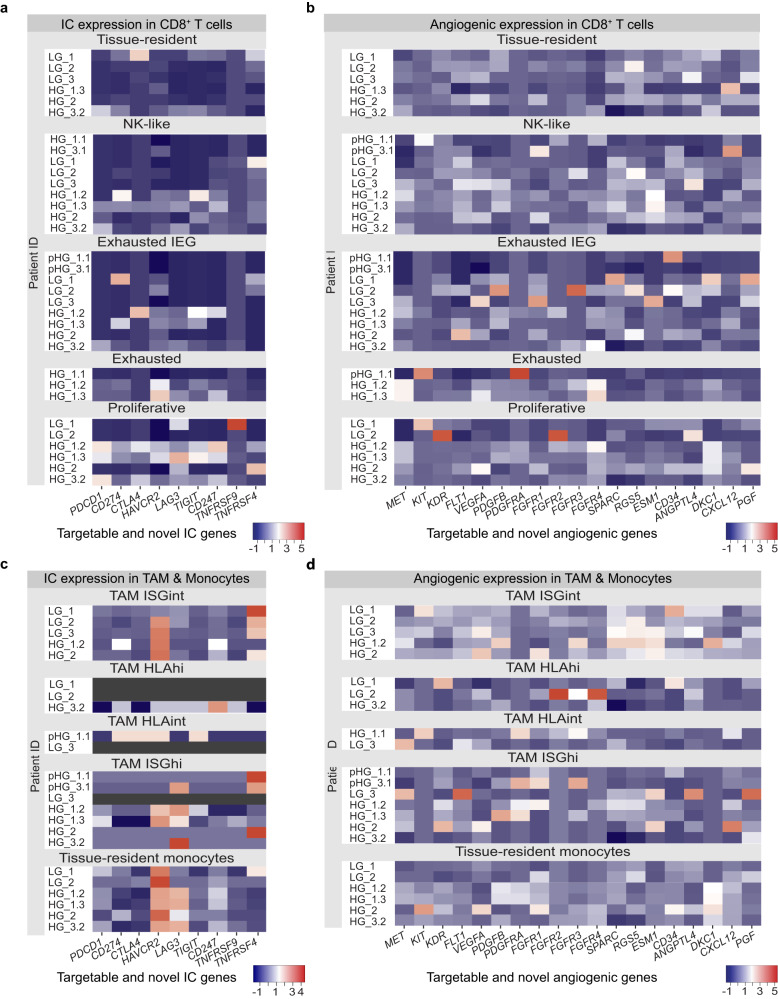
Targetable and novel IC and angiogenic gene expression within CD8^+^ T cells, TAM and monocytes. Targetable and novel ICs (**a**, **c**) demonstrated subtle relative increased expression within CD8^+^ proliferative cells for HG_1.2, HG_1.3 and HG_3.2 TMEs. Novel IC *HAVCR2* demonstrated a consistent increased expression within the exhausted tissue-resident monocytes across all TMEs. Novel IC *LAG3* demonstrated increased expression within exhausted TAM ISG^hi^ and tissue-resident monocytes in HG_1 and HG_3 TMEs. Targetable and novel angiogenic genes (**b**, **d**) demonstrated no consistent increased expression within CD8^+^ T cells, TAM and monocytes.

We investigated the CD8^+^ T cells, TAM and monocytes for the expression of targetable (*PD-1* or *PDCD1, CD274* or *PD-L1* and *CTLA4*) and novel (*CD247, LAG-3, PDCD1LG2* or *PD-L2, TIGIT, TNFRSF4 or OX40, TNFRSF9, TIM3 or HAVCR2*) IC genes. We determined the relative expression of targetable and novel IC genes within grouped CD8^+^ T cells (Fig. 3a), TAM and monocytes (Fig. 3c) across all pTME, LG and HG TMEs. No consistent expression of targetable IC genes was demonstrated. However, pro-tumour tissue-resident monocytes demonstrated a consistent increased relative expression of *HAVCR2* across all TMEs. Furthermore, pro-tumour TAM ISG^hi^ and tissue-resident monocytes within HG_1 and HG_3 TMEs demonstrated increased relative expression of novel IC *LAG3*.

Similarly, we investigated the relative expression of targetable (*MET*, *KIT*, *KDR* or *VEGFR1*, *FLT1* or *VEGFR2, VEGFA, PDGFB, PDGFRA*, *PDGFRB* and *FGFR1 to 4*) and novel (*ANGPTL4, CD34, CXCL12, DKC1, ESM1, PGF, RGS5* and *SPARC*) angiogenic genes within grouped CD8^+^ T cells (Fig. 3b), TAM and monocytes (Fig. 3d) across all pTME, LG and HG TMEs. No consistent increased expression of targetable or novel angiogenic genes was demonstrated within CD8^+^ T cells, TAM and monocytes across ccRCC TMEs.

## Discussion

In this study, we present the largest ST profile of immunophenotypes within para, low- and HG ccRCC TMEs. Consistent with previous bulk RNA-seq, single-cell RNA-seq (scRNA-seq) and single-nuclei RNA-seq (snRNA-seq) studies, we show abundant immune cell infiltration within the TME, dominated by T cells and monocytes^20–26^. Utilising published scRNA-seq and T-cell receptor sequencing data^26^, we show fine granular sub-typing of the CD8^+^ T cells, TAM and monocytes to non-exhausted/anti-tumour and exhausted/pro-tumour states. We show non-exhausted CD8^+^ T cells and anti-tumour TAM within all LG and HG_2 ccRCC tumour/immune regions. In contrast, within two of three HG ccRCC specimen (e.g., HG_1 and HG_3), we show predominantly exhausted CD8^+^ T cells and pro-tumour tissue-resident monocytes within the tumour/immune regions. In line with previous studies, we show a sub-population of exhausted CD8^+^ T-cell sub-types expressing stem-like progenitor (*TCF7*). Both sub-populations of exhausted and non-exhausted CD8^+^ T-cell sub-types express immunomodulatory (*ENTPD1*) genes^27–29^. The pro-tumour TAM and monocyte sub-types express an anti-inflammatory gene signature^28,30^. Similarly, *TREM2*, an established marker for post-surgical recurrence of ccRCC^30^, shows higher expression in a large proportion of the pro-tumour TAM and tissue-resident monocytes for patient HG_3 with recurrent ccRCC. Furthermore, within distinct tumour/immune admixed regions, the tissue-resident monocytes surround the exhausted CD8^+^ T cells, suggestive of anti-inflammatory activity and/or an exclusion zone along the tumour margins in HG TME, as identified in other studies^26,31,32^. Importantly, we demonstrate a singular exhausted/pro-tumour or non-exhausted/anti-tumour immune cell states within individual specimens, suggesting that lymphoid and myeloid cells behave in unison, possibly through cellular interactions between the immune cell sub-types, as identified in previous studies^23,26,33^.

Within the exhausted CD8^+^ T cells of the HG ccRCC TME, we show sub-populations expressing stem-like progenitor *TCF7*, suggestive of heterogeneity within the exhausted cell states and a capability of T cells to differentiate. It will be of interest to investigate within ex vivo studies, if these exhausted stem-like progenitors CD8^+^ T cells can be switched to an anti-tumour mode with ICI, as reported in other cancers^34–39^. The effectiveness of ICI is based on the expression of IC genes by infiltrating immune cells, as such we examined the expression of targetable and novel IC genes in grouped CD8^+^ T cells, TAM and monocytes across all samples. Targetable IC genes show no increase in expression within CD8^+^ T cells, TAM and monocytes, potentially explaining the limited efficacy of ICI^40^ observed in three adjuvant ICI trials^11–13^. However, these trial findings are in contrast with the Keynote-564 trial^41^. These conflicting trial outcomes of ICIs within intermediate high-risk ccRCC do not diminish the reported improved survival outcomes within the metastatic setting^42–44^. This further highlights a need for a precision oncological approach that integrates traditional histopathological and clinical information with systematic transcriptomics profiling.

Novel IC *HAVCR2* (or *TIM3*) shows consistently increased expression within the tissue-resident monocytes which dominated the HG TMEs. Traditionally, tissue-resident monocyte populations are considered reservoirs for macrophages that play key roles in immune defence and inflammatory events^45^. However, it is uncertain if tissue-resident monocytes have the plasticity to differentiate into macrophages and later TAM within the ccRCC TME. Mouse model studies have demonstrated that tissue-resident monocyte- and macrophage-derived TAM respond very differently to cancer treatment^46^. Further exploration of the fate of these tissue-resident monocytes in the HG ccRCC TME is needed to elucidate their response to ICI treatment. As such, we are interested in the results of the phase I and II anti-HAVCR2 trial (NCT02817633) in advanced solid tumours. Looking beyond potential IC targets, we extended our investigation to also include the expression of targetable and novel angiogenic genes. However, immune cells show no consistent increased expression of angiogenic genes, suggesting that the promotion of angiogenesis within the ccRCC TME may involve non-immune cells.

Limitations in this study include minimal multi-region sampling at a single timepoint from a modest cohort of consenting ccRCC patients and variable tumour stages within both the LG (LG_2, stage III) and HG (HG_3, stage I) ccRCC groups. These limits may have restricted complete molecular profiling of the heterogeneity and complexity of the intermediate high-risk ccRCC TME. Nonetheless, we show intra- and inter-patient heterogeneity within the identified immunophenotypes of the limited TME regions (i.e., pTME and TME) presenting at variable time points (i.e., localised lesion and advance recurrent and metastatic lesions). Therefore, our decision to use minimal multi-region sampling from six ccRCC patients for ST-seq preserves surgical margins, delivers uncompromised patient management, and still captures transcriptomic heterogeneity within the immunophenotypes present in the ccRCC TME. In future, the use of non-clinical mouse models of ccRCC can overcome these limits by allowing sampling from multi-regions and timepoints at similar tumour stage or a large clinical human cohort.

In summary, unbiased transcriptomic profiling identified heterogeneous immunophenotypes, without a consistent targetable IC expression profile, within intermediate high-risk ccRCC TMEs. Translating these findings in the future might result in identified immunophenotype characteristics in ccRCC that are favourable for ICI treatment and deliver precision oncology.

## Methods

### Patients and methods

We completed unbiased transcriptomic profiling of the immunophenotypes within individual ccRCC patients of differing clinical phenotypes. We focused on CD8^+^ T cells, TAM and monocytes within the TME due to their recognised effector and tolerogenic response, defined here as non-exhausted/anti-tumour and exhausted/pro-tumour states, respectively (Table 1). To characterise the non-exhausted or exhausted sub-types of CD8^+^ T cells and anti-tumour or pro-tumour sub-types of TAM and monocytes, we utilised recently published scRNA-seq datasets^26,47–52^.

### Patient characteristics

To profile the transcriptome within the ccRCC TME, we performed ST-seq using Visium Spatial Gene Expression (CG000239 Rev D, 2020 October, 10x Genomics, USA) on ten fresh frozen tissue sections collected in the operating theatre from six patients with ccRCC (*n* = 2 pTME, *n* = 4 LG and *n* = 4 HG) presenting at a tertiary referral hospital from June 2021 to January 2022 (Fig. 1a). Additional clinical characteristics of the ccRCC patients at presentation include patient LG_2 with a vena cava thrombus (VCT); patient HG_1 with pleural metastases; and patient HG_3 with a third recurrent lesion (Table 2). Furthermore, for patients HG_1 and HG_3, pTME tissue was collected from adjacent surgically normal margins. This study received ethics approval from Metro South Human Research Ethics Committee (Reference Numbers HREC/16/QPAH/353 and HREC/12/QPAH/125). All participants provided written informed consent to take part in the study.

### ST-seq method and analysis

In brief, the Visium ST-seq method (CG000239 Rev D, 2020 October, 10x Genomics, USA) involved the use of microarrayed glass slides with 55 µm spots (or ST-spots) containing oligonucleotides with a sequence of deoxythymine (oligo-dT) and unique spatial barcodes printed within capture arrays. Thin 8 µm cryosections were placed within a capture array overlaying the ST-spots^53^. Next, the tissue sections were stained by haematoxylin and eosin (H&E) and imaged on an Axio Z1 slide scanner (Zeiss). Afterwards, the same tissue sections were permeabilised to release mRNA. These mRNA were captured by underlying ST-spots, and complimentary DNA libraries incorporating the spatial barcodes were synthesised. All libraries were loaded at 1.8 pM and sequenced using a Mid output reagent kit (Illumina) on a NextSeq500 (Illumina) instrument. Sequencing was performed using the following protocol: Read1 - 28 bp, Index1 - 10 bp, Index2 - 10 bp, Read2 - 120 bp. After sequencing the genes were mapped to the H&E images to generate spatially resolved transcriptional profiles (Fig. 1b)^54^.

The generated ST-seq datasets were processed and analysed using STUtility (v0.1.0)^55^ and Seurat (v4.1.0) R packages^56,57^. We confirmed the quality of the captured transcriptome using the following cut-offs: >50 genes per ST-spot, >100 unique molecular identifiers (UMI) per gene, ≥500 nCount per ST-spot, >500 nFeature per ST-spot and <30% mitochondrial genes per ST-spot. Then, we merged all the individual ST-seq datasets and removed batch effects due to individual patient samples using the SCTransformfunction in Seurat^58^. With this merged ccRCC ST-seq dataset, Louvain clustering was performed with the most stable cluster resolution (res 0.4). These clusters were then annotated as immune and non-immune using published kidney (healthy^47,48^, inflammed^49,50^ and renal tumour^51^) and tumour immune atlas^52^ scRNA-seq datasets.

Next, immune ST-spots were selected for detailed immune cell sub-typing using a recent publicly available scRNA and T-cell receptor (TCR)^26^ sequencing dataset from ccRCC patients that were ICI naïve or exposed. In brief, the scRNA/TCR-seq dataset contained curated transcriptome signatures for exhausted or non-exhausted lymphoid and pro- or anti-tumour myeloid cell sub-types. Based on these signatures, we annotated the cell states of the T cells, macrophages and monocytes using the Semi-supervised Category Identification and Assignment (SCINA) algorithm^59^. In brief, this method leverages previously established gene signatures in a semi-supervised model using an expectation–maximisation (EM) algorithm. Next, we focused on the CD8^+^ T cells, TAM and tissue-resident monocytes (Table 1). CD8^+^ T-cell sub-types with an exhausted state are: CD8^+^ exhausted, CD8^+^ proliferative, CD8^+^ exhausted immediate-early genes (CD8^+^ exhausted IEG); and non-exhausted state are: CD8^+^ tissue-resident and CD8^+^ NK-like. Myeloid cell sub-types with a pro-tumour state are: TAM with human leucocyte antigen DR (HLA-DR) high expression (TAM HLA^hi^), TAM with HLA-DR intermediate expression (TAM HLA^int^), TAM with interferon signalling genes high expression (TAM ISG^hi^) and CD14^+^/16^+^ monocytes termed tissue-resident monocytes; and anti-tumour state TAM ISG intermediate expression (TAM ISG^int^). This classification of exhausted/pro-tumour or non-exhausted/anti-tumour immune cell states is not static. Indeed, a spectrum of immune cell states is being recognised^60^. However, for brevity, here we utilised exhausted or non-exhausted states for five CD8^+^ T-cell sub-types and pro- or anti-tumour states for five TAM and tissue-resident monocyte sub-types identified within our ST-seq datasets (Table 1). Linear modelling was used to test for differences in cell type composition between grades with the propeller method implemented within the speckle R package^61^.

### Reporting summary

Further information on research design is available in the Nature Research Reporting Summary linked to this article.

### Supplementary information

Supplementary Material

Reporting Summary

## Data Availability

Raw Sequencing and Spaceranger processed files have been deposited in ArrayExpress (Annotare2.0) data repository with the accession number E-MTAB-12767. Post-analysis files generated during the analysis of this project are available through the Zenodo repository (10.5281/zenodo.7619249).
