# Supplementary material for: High risk clear cell renal cell carcinoma microenvironments contain protumour immunophenotypes lacking specific immune checkpoints
Source: NPJ Precis Oncol. 2023 Sep 11;7:88. doi: 10.1038/s41698-023-00441-5 (PMC10495390; doi:10.1038/s41698-023-00441-5)
Supplement: Supplementary file 1 — Supplementary Material [file 41698_2023_441_MOESM1_ESM.pdf]

Supplementary Material

Supplementary Table 1. A comparison of the proportion of immune infiltrate, immune cell types, CD8$^+$ T, TAM and monocyte sub-types within the ccRCC TMEs. Statistical significance of the identified proportions of immune infiltrates within the TMEs (ANOVA), immune cell types, CD8$^+$ T cell sub-types and TAM/Monocyte sub-types (pair-wise tests).

	All grades (p.value)	HG vs LG TME (p.value)	HG vs pTME (p.value)	LG vs pTME (p.value)
**Immune infiltration within the TMEs**				
**Immune/non-immune**	2.90^{-10}	0.007	9.00^{-7}	0.058
**Immune cell types**				
**CD4$^+$ T cells**	2.8^{-4}	1.70^{-5}	7.10^{-06}	0.032
**CD8$^+$ T cells**	7.1^{-3}	6.20^{-5}	0.016	0.054
**Macrophages**	1.5^{-2}	5.3^{-3}	0.16	0.1
**Monocytes**	0.26	0.38	0.33	0.28
**CD8$^+$ T cells sub-types**				
**CD8$^+$ Tissue-resident**	3.1^{-4}	1.00^{-4}	0.38	1.4^{-3}
**CD8$^+$ NK-like**	0.13	0.51	0.087	0.17
**CD8$^+$ Exhausted IEG**	0.021	8.3^{-3}	0.7	1.8^{-3}
**CD8$^+$ Exhausted**	0.083	0.29	0.73	0.14
**CD8$^+$ Proliferative**	8.9^{-4}	2.7^{-4}	0.031	0.14
**TAM/Monocytes sub-types**				
**TAM ISG^{int}**	9.50^{-13}	2.8^{-4}	0.12	8.4^{-4}
**TAM HLA^{hi}**	0.3	0.41	0.13	0.49
**TAM HLA^{int}**	1.90^{-5}	0.046	3.1^{-4}	5.8^{-3}
**TAM ISG^{hi}**	6.4^{-4}	0.15	8.5^{-3}	5.9^{-3}
**Tissue-resident monocytes**	7.30^{-11}	1.4^{-3}	5.40^{-5}	0.86

Supplementary Table 2. *TCF7* and *ENTPD1* genes within the high-grade CD8$^+$ T cell sub-types. Within the high-grade TMEs, we investigated the stem-like progenitor (*TCF7*) and immunomodulatory (*ENTPD1*) genes within each CD8$^+$ T cell sub-type. For each CD8$^+$ T cell sub-type, we determined both the percentage of the ST-spots and their relative expression of *TCF7* and *ENTPD1* genes. The highest relative expression of TCF7 was identified within sub-populations of CD8$^+$ exhausted IEG and CD8$^+$ exhausted T cells within HG_1.2 and HG_1.3. The highest relative expression of *ENTPD1* was identified within sub-populations of exhausted CD8$^+$ proliferative T cells in HG_1.3 and HG_3.2, and non-exhausted CD8$^+$ tissue-resident T cells in HG_2.

		TCF7		*ENTPD1*	
	**CD8+ T cell sub-type**	**% ST-spots**	**Relative expression**	**% ST-spots**	**Relative expression**
**HG_1.2**	Tissue-resident				
	NK-like	0.13	-0.39	0.33	-0.44
	Exhausted IEG	0.08	0.69	0.10	-0.44
	Exhausted			0.18	-0.44
	Proliferative	0.18	-0.39	0.15	-0.44
**HG_1.3**	Tissue-resident				
	NK-like			0.14	-0.44
	Exhausted IEG	0.14	-0.39	0.14	-0.44
	Exhausted	0.11	2.85	0.05	-0.44
	Proliferative	0.15	-0.39	0.27	1.78
**HG_2**	Tissue-resident	0.03	-0.39	0.13	2.77
	NK-like	0.05	-0.39	0.05	-0.44
	Exhausted IEG				
	Exhausted				
	Proliferative			0.20	-0.44
**HG_3.2**	Tissue-resident				
	NK-like	0.03	-0.39	0.18	-0.44
	Exhausted IEG				
	Exhausted	0.04	-0.39	0.13	-0.44
	Proliferative	0.03	-0.39	0.13	0.24

Supplementary Figure 1. Anti-inflammatory gene signature in TAM and monocyte sub-types within the high-grade ccRCC TME. The anti-inflammatory gene signature demonstrated consistent expression levels across TAM and monocytes within the HG TMEs. However, triggering receptor expressed on myeloid cells 2 (*TREM2*, orange) demonstrated higher expression levels within pro-tumourTAM HLA^{hi} and TAM ISG^{hi} in HG_3 TME. The anti-inflammatory gene signature includes interferon-responsive (*IFI27*), cysteine cathepsins (*CTSL, CTSS*), complement-related (*C1QA, C1QB, C1QC, SERPING1*), lipid transport-related (*APOE, PLTP, TREM2*) and HLA class I (*HLA-A, HLA-B, HLA-C, HLA-E, HLA-F* and *HLA-G*) and II (*HLA-DPA1, HLA-DQA1, HLA-DRA, HLA-DMB* and *HLA-DOA*) genes.

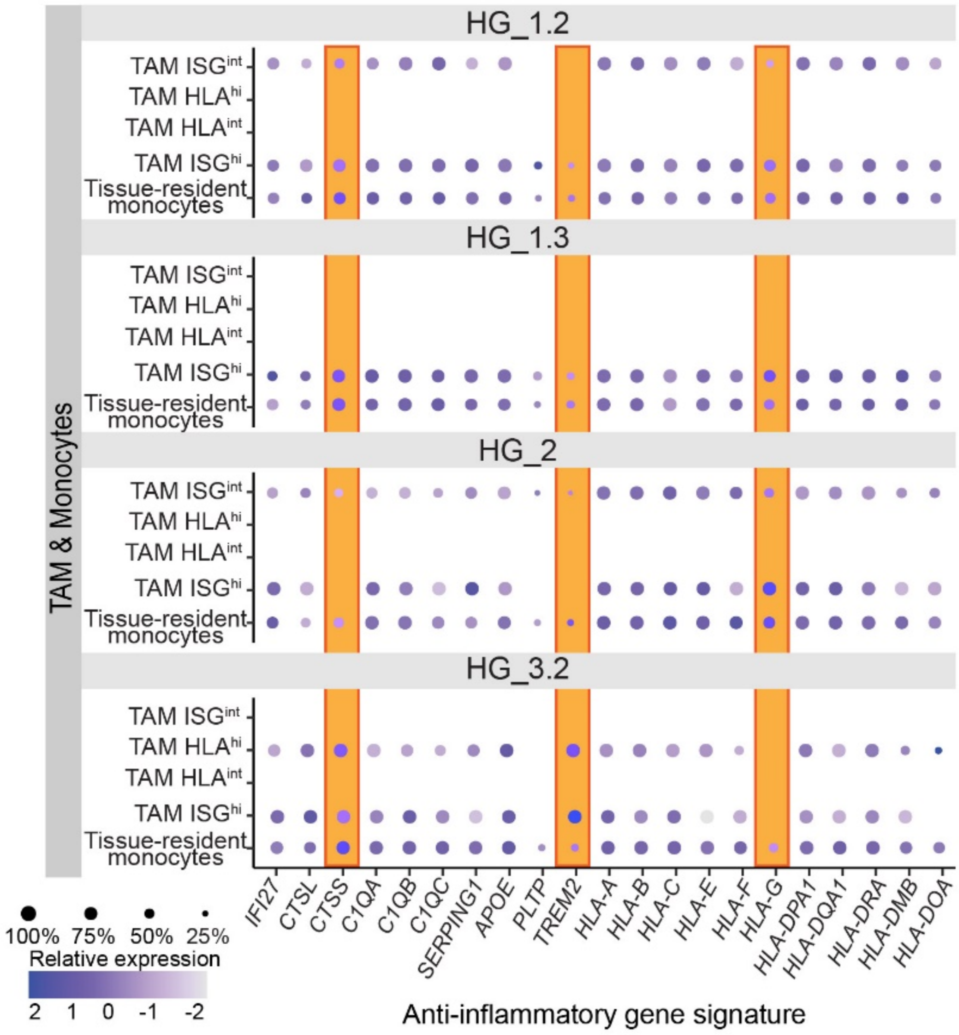

Supplementary Figure 2. CD8$^+$ T cells, TAM and Monocytes mapped within the TME. Exhausted CD8$^+$ T cell and pro-tumour TAM and monocyte sub-types are mapped within respective tissue sections annotated based on histological features of tumour/immune, non-tumour, fibrotic/tumour/immune, fibrotic, scarred regions with immune infiltrate, immune nests and oedema. Exhausted CD8$^+$ T cell and pro-tumour TAM and monocyte sub-types were absent within the pHG_1.1 and pHG_3.1 but abundant within respective HG_1.3 and HG_3.2 tumour/immune regions. However, all LG and HG_2 TMEs demonstrated abundant non-exhausted CD8$^+$ T cell and anti-tumour TAM sub-type within the tumour/immune regions.

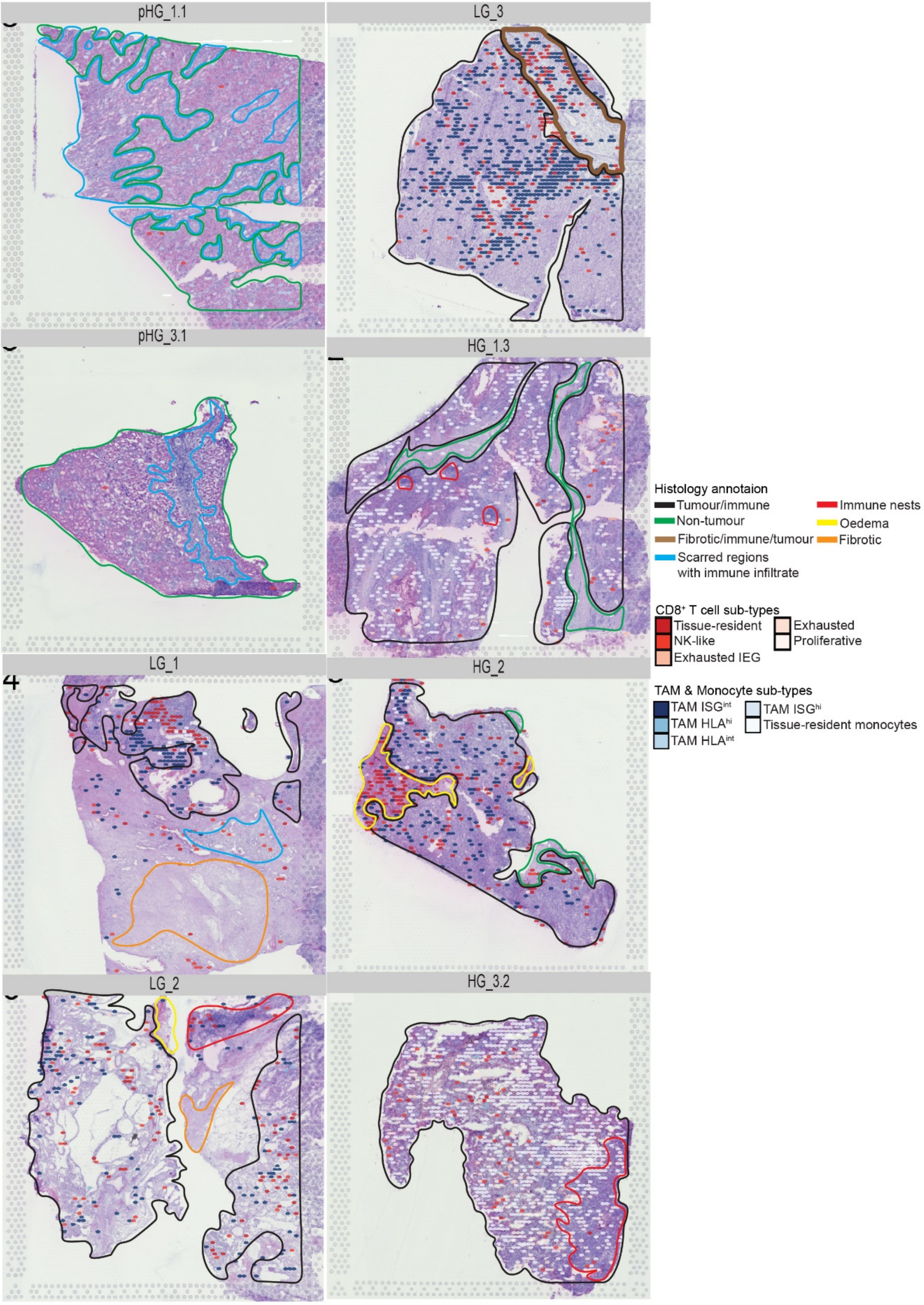
pHG_1.1
LG_3
pHG_3.1
HG_1.3
LG_1
HG_2
LG_2
HG_3.2
4
Histology annotaion
Tumour/immune
Non-tumour
Fibrotic/immune/tumour
Scarred regions with immune infiltrate
Immune nests
Oedema
Fibrotic
CD8+ T cell sub-types
Tissue-resident
NK-like
Exhausted IEG
Exhausted
Proliferative
TAM & Monocyte sub-types
TAM ISG int
TAM HLA hi
TAM HLA int
TAM ISG hi
Tissue-resident monocytes
