# Supplementary material for: High risk clear cell renal cell carcinoma microenvironments contain protumour immunophenotypes lacking specific immune checkpoints
Source: NPJ Precis Oncol. 2023 Sep 11;7:88. doi: 10.1038/s41698-023-00441-5 (PMC10495390; doi:10.1038/s41698-023-00441-5)
Supplement: Supplementary file 2 — Reporting Summary [file 41698_2023_441_MOESM2_ESM.pdf]

nature portfolio

Corresponding author(s): Andrew J. Mallett

Last updated by author(s): Jun 10, 2023

Reporting Summary

Nature Portfolio wishes to improve the reproducibility of the work that we publish. This form provides structure for consistency and transparency in reporting. For further information on Nature Portfolio policies, see our Editorial Policies and the Editorial Policy Checklist.

Statistics

For all statistical analyses, confirm that the following items are present in the figure legend, table legend, main text, or Methods section.

n/a	Confirmed	
☐	☒	The exact sample size (*n*) for each experimental group/condition, given as a discrete number and unit of measurement
☐	☒	A statement on whether measurements were taken from distinct samples or whether the same sample was measured repeatedly
☐	☒	The statistical test(s) used AND whether they are one- or two-sided<br>*Only common tests should be described solely by name; describe more complex techniques in the Methods section.*
☐	☒	A description of all covariates tested
☐	☒	A description of any assumptions or corrections, such as tests of normality and adjustment for multiple comparisons
☐	☒	A full description of the statistical parameters including central tendency (e.g. means) or other basic estimates (e.g. regression coefficient) AND variation (e.g. standard deviation) or associated estimates of uncertainty (e.g. confidence intervals)
☐	☒	For null hypothesis testing, the test statistic (e.g. F, t, r) with confidence intervals, effect sizes, degrees of freedom and P value noted<br>*Give P values as exact values whenever suitable.*
☒	☐	For Bayesian analysis, information on the choice of priors and Markov chain Monte Carlo settings
☐	☒	For hierarchical and complex designs, identification of the appropriate level for tests and full reporting of outcomes
☐	☒	Estimates of effect sizes (e.g. Cohen's *d*, Pearson's *r*), indicating how they were calculated

Our web collection on statistics for biologists contains articles on many of the points above.

Software and code

Policy information about availability of computer code

Data collection

In brief, the ST-seq method (CG000239 Rev D, 2020 October, 10x Genomics, USA) involved the use of microarrayed glass slides with 55 µm spots (or ST-spots) containing oligonucleotides with a sequence of deoxythymine (oligo-dT) and unique spatial barcodes printed within capture arrays. Thin 8 µm cryosections were placed within a capture array overlaying the ST-spots. Next, the tissue sections were stained by haematoxylin and eosin (H&E) and imaged on an Axio Z1 slide scanner (Zeiss). Afterwards, the same tissue sections were permeabilised to release mRNA. These mRNA were captured by underlying ST-spots, and complimentary DNA libraries incorporating the spatial barcodes were synthesised. All libraries were loaded at 1.8 pM and sequenced using a Mid output reagent kit (Illumina) on a NextSeq500 (Illumina) instrument. Sequencing was performed using the following protocol: Read1 - 28bp, Index1 - 10bp, Index2 - 10bp, Read2 - 120bp. After sequencing the genes were mapped to the H&E images to generate spatially resolved transcriptional profiles (Fig. 1B)25.

The generated ST-seq datasets were processed and analysed using STUtility (v0.1.0)26 and Seurat (v4.1.0) R packages27,28. We confirmed the quality of the captured transcriptome using the following cut-offs: > 50 genes per ST-spot, > 100 unique molecular identifiers (UMI) per gene, ≥ 500 nCount per ST-spot, > 500 nFeature per ST-spot and < 30% mitochondrial genes per ST-spot. Then, we merged all the individual ST-seq datasets and removed batch effects due to individual patient samples using the SCTransformfunction in Seurat29. With this merged ccRCC ST-seq dataset, Louvain clustering was performed with the most stable cluster resolution (res 0.4). These clusters were then annotated as immune and non-immune using published kidney (healthy18,19, inflammed20,21 and renal tumour22) and tumour immune atlas23 scRNA-seq datasets.

Data analysis

Next, immune ST-spots were selected for detailed immune cell sub-typing using a recent publicly available scRNA and T cell receptor (TCR)24 sequencing dataset from ccRCC patients that were ICI naïve or exposed. In brief, the scRNA/TCR-seq dataset contained curated transcriptome signatures for lymphoid and myeloid cell sub-types, and their non-exhausted or exhausted states. Based on these signatures, we annotated the cell states of the T cells, macrophages and monocytes using the Semi-supervised Category Identification and Assignment (SCINA)

algorithm30. In brief, this method leverages previously established gene signatures in a semi-supervised model using an expectation–maximization (EM) algorithm. Next, we focused on the CD8+ T cells, TAM and tissue-resident monocytes (Table 1). Immune cell sub-types with an exhausted state include: CD8+ exhausted, CD8+ proliferative, CD8+ exhausted immediate-early genes (CD8+ exhausted IEG), TAM with human leukocyte antigen DR (HLA-DR) high expression (TAM HLAhi), TAM with HLA-DR intermediate expression (TAM HLAint), TAM with interferon signalling genes high expression (TAM ISGhi) and CD14+/16+ monocytes termed tissue-resident monocytes. Immune cell sub-types with a non-exhausted state include: CD8+ tissue-resident, CD8+ NK-like and TAM ISG intermediate expression (TAM ISGint) cells. This classification of exhausted or non-exhausted immune cell states is not static. Indeed, a spectrum of immune cell states are being recognised31. However, for brevity, here we utilised exhausted or non-exhausted states to classify the ten immune cell sub-types identified within our ST-seq datasets (Table 1).

For manuscripts utilizing custom algorithms or software that are central to the research but not yet described in published literature, software must be made available to editors and reviewers. We strongly encourage code deposition in a community repository (e.g. GitHub). See the Nature Portfolio guidelines for submitting code & software for further information.

Data

Policy information about availability of data

All manuscripts must include a data availability statement. This statement should provide the following information, where applicable:

- Accession codes, unique identifiers, or web links for publicly available datasets
- A description of any restrictions on data availability
- For clinical datasets or third party data, please ensure that the statement adheres to our policy

Raw Sequencing and Spaceranger processed files have been deposited in ArrayExpress (Annotare2.0) data repository with the accession number XXXXXXX (This process is under way and we are currently liaising with ArrayExpress staff to finalise the upload; it will be finalised and completed prior to potential publication). Post-analysis files generated during the analysis of this project are available through the Zenodo repository (10.5281/zenodo.7619249). [Currently Embargoed Access until published]

Research involving human participants, their data, or biological material

Policy information about studies with human participants or human data. See also policy information about sex, gender (identity/presentation), and sexual orientation and race, ethnicity and racism.

Reporting on sex and gender	Biological Sex is reported. Both Male and Female participants included.
Reporting on race, ethnicity, or other socially relevant groupings	Not reported as not recorded in the study records for participants.
Population characteristics	Age, biological sex, tumour characteristics, comorbidities, treatment, follow up
Recruitment	Consecutive patients presenting to a tertiary referral hospital (Princess Alexandra Hospital, Brisbane, Australia), June 2021 to Jan 2022. All participants provided written informed consent to take part in the study
Ethics oversight	This study received ethics approval from Metro South Human Research Ethics Committee (Reference Numbers HREC/16/QPAH/353 and HREC/12/QPAH/125).

Note that full information on the approval of the study protocol must also be provided in the manuscript.

Field-specific reporting

Please select the one below that is the best fit for your research. If you are not sure, read the appropriate sections before making your selection.

☒ Life sciences ☐ Behavioural & social sciences ☐ Ecological, evolutionary & environmental sciences

For a reference copy of the document with all sections, see nature.com/documents/nr-reporting-summary-flat.pdf

Life sciences study design

All studies must disclose on these points even when the disclosure is negative.

Sample size	Six patients, convenience sample
Data exclusions	No data exclusions
Replication	Intersample comparisons and comparison to public datasets
Randomization	N/A
Blinding	N/A

Reporting for specific materials, systems and methods

We require information from authors about some types of materials, experimental systems and methods used in many studies. Here, indicate whether each material, system or method listed is relevant to your study. If you are not sure if a list item applies to your research, read the appropriate section before selecting a response.

Materials & experimental systems

n/a	Involved in the study
☒	☐ Antibodies
☒	☐ Eukaryotic cell lines
☒	☐ Palaeontology and archaeology
☒	☐ Animals and other organisms
☐	☒ Clinical data
☒	☐ Dual use research of concern
☒	☐ Plants

Methods

n/a	Involved in the study
☒	☐ ChIP-seq
☒	☐ Flow cytometry
☒	☐ MRI-based neuroimaging

Clinical data

Policy information about clinical studies

All manuscripts should comply with the ICMJE guidelines for publication of clinical research and a completed CONSORT checklist must be included with all submissions.

Clinical trial registration	N/A not a trial
Study protocol	The underpinning clinical study protocols are available at https://dora.health.qld.gov.au/qldresearchjspui/cris/project/pj09498 and https://dora.health.qld.gov.au/qldresearchjspui/cris/project/pj12251
Data collection	Primary Clinical Data collected from source patient records at Princess Alexandra Hospital in line with participant consent.
Outcomes	Observational only, restricted to progression or incidence of local recurrence or metastasis
